# Injuries in the Present War

**Published:** 1915-05

**Authors:** W. W. Quinton

**Affiliations:** Buffalo, N. Y.


// BUFFALO MEDICAL JOURNAL

Yearly Volume 70 MAY, 1915 No. 10

### ORIGINAL ARTICLES

The right is reserved to decline papers not dealing with practical medical and surgical subjects, and such as might offend or fail to interest readers. Contributors are solely responsible for opinions, methods of expression and revision of proof.

### Injuries In The Present War.

By W. W. QUINTON, M. D., Buffalo, N. Y.

A few short years ago we thought we knew all there was to know about modern arms and projectiles and their effect on the human body. Our studies of the Spanish-American war and the Russo-Japanese war revealed much, but it seems as if the present war must revolutionize all our ideas on this subject.

This is not due so much to a marked change in armament, as it is to changed conditions in the conduct of the war; conditions unlooked for and regarded as impossible. The fighting is being conducted at point-blank range and the combatants are being subjected to the full effect of the 20th Century engines of war.

We have to consider the effect of rifle bullets, shells, (percussion and shrapnel), bombs fired from trench mortars, arrows rained down from aeroplanes, machine guns, pistol balls, and even bayonet.

The rifle bullets are, for the most part, about 30 caliber, light, sharp pointed, and with an extremely high velocity—nearly 3,000 feet per second muzzle velocity. The French bullet is said to be made of solid brass; the German, more like our own, has a jacket of cupro-nickle steel and a hardened lead core.

The percussion shells burst on impact, are filled with a high explosive charge and have a thick steel case. Their explosion is followed by the distribution of ragged pieces of steel in all directions, with terrific force, causing the most frightful wounds. The shrapnel have a time fuse which is cut by the gunner to burst in a given number of seconds. The fuse is lighted by the powder charge which propels the projectile, when the piece is fired. The shrapnel are built up; a plate of friable iron on which rest a number of hardened lead balls, caliber 50; another iron plate, then another layer of balls, and so on until the case is filled; the fuse forming the point of the shell. They contain a powder charge just sufficient to burst the case, which is about 3-16ths of an inch in thickness. When the shell bursts the friable iron plates are broken by the explosion and, with the balls, make about 300 fragments which are propelled forward, cone-shaped, at the original velocity of the shell. The bombs are fired by trench mortars at close range. They are about 7 inches in diameter and contain only the bursting charge of explosive. On bursting the outside casing is torn to bits and the steel fragments are sent flying in all directions. The arrows from aeroplanes have a velocity sufficient to drive them through a man's body from his head to his feet. The pistol balls are of small caliber, from 30 to 38, some of them made of hardened lead and others with steel jackets, similar to the rifle bullets.

Much has been said about the use of ''mushroom'' bullets by the combatants in the various armies. I think it may be safely said that none of these armies have used, are using, or ever will use bullets of this description. There are several factors that enter into the question and mislead individuals into thinking that they have to deal with wounds from ''mushroom'' bullets. Explosive effects do occur in the use of the high power rifles, and the causes may be divided into three classes:

1. High velocity, causing extensive comminution of bones when struck at a right angle.

2. Instability of flight and a tendency on the part of the bullet to revolve around a transverse axis. Unless impact occurs with almost the exact tip, a rapid revolution of the bullet on its axis occurs and it is driven sideways through the tissues, producing a large wound of exit. This is particularly true of the German bullet.

3. Ricochet. This is where a bullet first strikes some object and is deflected, entering the body of the soldier in a greatly deformed condition and causing an extensive wound.

When men are only a few yards apart, as they are in the present struggle, the most typical explosive effects are produced.

Rifle bullets may pass through cancellous bony tissue with a true perforating effect; when they strike the shaft of a bone, comminution and splintering effects are produced to an advanced degree. These bullets do not, as a rule, carry clothing or other foreign bodies into the wound and generally the shock from such wounds is not great. The shrapnel balls, being of large caliber and of a slower velocity are apt to carry foreign substances into the wound, but in case they do not the healing is uneventful, with small discomfort to the patient, even though the ball may not have been extracted.

The shell wounds, that is those caused by fragments of the steel case, are the worst and the most common—occurring in a proportion of two shell wounds to one of any other character.

An interesting fact is revealed by the use of the X-Ray in wounds, where the jacket of the bullet has been stripped off. Here the lead of the core is scattered and appears in the X-Ray picture like the smooch of a soft lead pencil. In operating, one can follow the track of the bullet by the color of the muscle. After hitting a bone, bone fragments and lead particles show up in the picture in perfect radiating lines, with a "tail of a comet" effect. If the bullet splits, two paths will be seen of a like character. The shrapnel bullet does not do this.

Some of the results of this war will be an immense number of stiff joints, flail joints and badly united fractures, with consequent loss of function, which will tax the ingenuity of the orthopaedic surgeons.

It is apparent that first aid dressings applied in this war have not had the desired result in preventing sepsis. The reason is apparent. In the Spanish-American War, the men went into battle in a comparative cleanly condition and wound infection was reduced to a minimum. In the Russo-Japanese war, we read that the Japanese required their soldiers to bathe their bodies and change their clothing before they engaged the enemy. In Europe, we see men fighting in trenches half filled with mud and water and slime, filthy dirty, unable to change their clothing for weeks at a time, unable even to remove a portion of their clothing, and bathing, naturally, an impossibility. Is it any wonder then, that nearly every case is infected when it arrives at the base hospital? Septic wounds of all descriptions; septic compound fractures with extensive laceration; femurs with 3 inch shortening and so badly infected that it is next to impossible to reduce them? It may well be believed that aseptic treatment in such cases has been found a failure and that antiseptic measures preceded by a thorough mechanical scrubbing and copious lavage have been found necessary.

Gaseous gangrene from soil infection has been of frequent occurrence in the present war. Some localities seem to offer greater danger of this dread complication than others and it seems to be especially prevalent in Northern France. The causative organism is anaerobic and for this reason we do not get this form of gangrene when the wounds are freely open.

It is a matter of interest that the bacillus may be present in open wounds existing as a harmless saprophite. It is in the small, deep, tortuous variety of injury where the infection causes trouble. It is well to remember that the germ is of the spore-bearing variety and prolonged boiling of instruments in antiseptic solutions, such as carbolic acid 1-20, and the destruction of all articles of clothing that come in contact with the wound are necessary to avoid further inoculation of patients.

If a case occurs, we find swelling, very severe pain, a quantity of blood stained serum exuding from the wound, a characteristic and very offensive odor, the tissues surrounding the wound purplish or slate colored and very speedily becoming black. Beyond the area of discoloration one gets the crepitation of the tissues due to the gas formed by the bacillus. Tight bandaging may predispose by interfering with the circulation and the free discharge of serum from a wound. If the infection is not too severe, the wound may be freely opened, foreign bodies removed, and irrigation with peroxide of hydrogen used. If the infection is more severe, amputation performed high above the infected area may save the patient. Too often a few hours suffice to bring about the dissolution of the patient. We had our experiences with the "gas bacillus" in the Philippines, where we learned to appreciate the gravity of the infection.

For the location of bullets in the tissues, the British are using the telephonic bullet probe to a certain extent. The procedure is quite simple—no battery is required. One wire is grounded on the patient, using a metal or carbon plate on a saline pad, and the other wire is attached to the probe.

With all the modern army surgeon has to contend with in the way of newer and more deadly devices to take away human life, we must admit that the results of surgical treatment have never been better. The human frame will stand a lot of manhandling, and I should like to cite just a few cases to prove this.

In the Spanish-American war, as I have said, our first-aid dressings were eminently successful. I gave first-aid treatment to the first man who drew a pension as a result of physical disability from wounds in action. He was struck by a fragment of shell which removed half of his nose, half of his upper jaw, half of his tongue, half of his lower jaw and his chin. The wound was packed with gauze to stop the hemorrhage, and he was transported to the rear. After his return to the States, plastic operations were performed with excellent cosmetic effect.

A man struck in the back below the left scapula by several shrapnel balls which carried a section of his blue flannel shirt into the wound, received first-aid and was transported to the rear. I saw him some years afterwards. He was in good health but complained that the shrapnel balls, which had never been extracted, had worked their way, well down towards his left groin!

Our abdominal cases were transported to the base hospitals and operated upon with tremendous mortality. Abdominal wounds will not stand much interference under field conditions, and will all die if operated. The best that can be done is to keep the patient in a sitting position without food until he arrives at some place where operative procedures can be carried out with some hope of success. Expectant treatment seems to give the best results.

At the American Woman's War Hospital, Paignton, England, a number of interesting cases are reported. Here are a few of them:

A man with the upper half of the humerus and the short head of the biceps shot away, had a resection, with the result that he can write as well as ever—however he cannot raise his arm above his head.

Eleven men were on a gun crew when a German shrapnel burst amongst them. Five were killed and six wounded. One patient had: one wound of the left leg and seven large wounds of the right leg with a compound fracture of both bones. One fragment of steel was removed from under one of his toe nails soon after admission, and a little later the barber, while shaving him, nicked his razor on a good-sized sliver of steel which he extracted through a pimple-like opening in his cheek. This man had a temperature of 104 degrees for some days but recovered.

Another patient is doing well with a bullet wound of the liver, causing a biliary fistula forward and back; an empyema; healed wound of the shoulder, and septic wounds of one hand and one foot! Enough for one man! When we consider that wounds of the solid viscera are nearly always fatal, we may marvel more at his good-fortune.

A patient with part of the sacrum blown away and seven inches of the rectum exposed, is recovering.

Another with both trochanters bare and an infected wound of the lower part of the thigh, had the thigh amputated with a resulting good stump and is also recovering.

A case reported by another authority, was struck simultaneously by a number of machine gun bullets. One wound in the head, one oblique wound of the upper jaw, a bullet through the chin, one through the right arm and one in the left wrist resulted. Two others wounded his helmet and boot. He lived to tell the tale.

In spite of the terrible character of the war, there are some cheering statistics. For instance, here are some of the reports from hospitals in England:

| | |
|---|---|
| Oxford Base Hospital | 3,000 cases, 12 deaths |
| Cambridge Hospital | 3,500 cases, 15 deaths |
| Paignton Hospital | 700 cases, 1 death |

From a French report:

| | |
|---|---|
| Wounded able to return at once | 54.5 per cent. |
| Wounded sent away for convalescence | 24.5 per cent. |
| Wounded under hospital treatment | 17.4 per cent. |
| Wounded discharged for disability | 1.46 per cent. |
| Died | 3.48 per cent. |

These figures are most favorable and represent the triumph of modern surgical methods, applied under the most adverse circumstances, over the injuries inflicted by the newly invented carriers of death.

I would like to acknowledge the assistance received from the reports of Dr. Robert W. Hinds, late of this city and now serving in The Woman's War Hospital, Paignton, England.

---

**Treatment of Manifest Tetanus.** Manheimer-Gommes, Bull. et Mem. de la Soc. de Med. de Paris, Dec. 11, 1914. The author does not insist on the extraction of the foreign body nor on the daily antiseptic lavage of the wound, with hydrogen peroxid, alkaline solutions, concentrated permanganate of potassium, etc. (Note. From the context, we are inclined to think that the use of the word insist is idiomatic and opposite to the English meaning, implying that he does not consider it necessary to dwell upon these methods). He prefers hypodermatic or intravenous administration of medicines, the buccal method being somewhat unreliable, the rectal and especially the spinal methods troublesome, particularly to the patient. 20-30 c.c. of serum, daily, seems to act as well as 50 c.c. and even the economy must be considered in military practice. He has not noted unfavorable results, except a slight and transient cardiac depression, with cyanosis. 2% phenol solutions, 1-3 c.c. a day, not over 1.50 c.c. at an injection are painless and no accidents arise from lack of aseptic precautions which are not always possible at the front. Chloral (by mouth or rectum) not over 15 grams a day. He has used hyoscyamine and morphine but no other narcotics. Constipation, retention of urine and bronchoplegia—paralysis of the smooth muscle of the branches and radicles of the bronchial tubes—with its tendency to bronchial infection, he considers due mainly to morphine and avoids its use.

---

**Sawdust,** excluding large fragments by No. 8 sieve and fine dust by No. 40, packed in gauze and sterilized by heat, is being extensively used as a dressing in the European War.

